# A Subject of Overwhelming Importance

**Published:** 1920-02-21

**Authors:** 


### THE ECONOMICS OF NURSING.

### A Subject of Overwhelming Importance.

UNDER this comprehensive title I have contributed many articles to THE HOSPITAL. Those articles have been more or less at haphazard, and topical, a fact of which I have always been painfully cognisant. The subject is overwhelmingly important, and really calls for a treatise based on scientific principles. The economics of nursing concern not only the nurse, who is directly and intimately affected, but they concern equally hospital committees, and they concern most of all the British nation.

It is a fact, not a little remarkable, that big men, statesmen for example, almost tremble if it is proposed to depart from tradition. The conservative tendency of our people is to regard what is, and what has been, rather than what must be. I was, for a long time, extremely anxious to have in writing a standard and authoritative opinion as to what a nurse's working day ought to be. Now I have it from the chairman of the "London." Anything short of a twelve hours' day is "impossible." It does not weary the nurse, and it makes possible the adequate attention by trained women to the wants of the nation's poor. In giving his views Lord Knutsford was not arguing only from the standpoint of a public hospital. He was, at the same time, considering the private employer. And I have no intention of suggesting that, from one point of view, he is wrong. Cheap labour appeals to everybody in the world, except the labourer. All classes of labour were cheap seven years ago compared with now, and, if we go back two or three generations, labourers were chattels and were sold like cattle. But the labourer is marching along, and, willy nilly, if you want him you must expand. There is no alternative. And yet, as I say, big men tremble at the thought of change. They are tied by tradition, and they appear to think that that which is wrong must be perpetuated, simply because it has always been wrong. The teaching profession is analogous. The schoolmaster has always been underpaid, with the result that the best men seek employment elsewhere. I am struck by a remark made in the Educational Supplement to the *Times*, current issue, on the supply of teachers. "It is no use," says the writer, "for the President of the Board of Education to make speeches saying that teaching cannot compete with other professions. It can, and it must, compete with other branches of the public service!" Quite so. Why should it not? Otherwise we shall have an inefficient army of men and women teaching the rising generation what it ought to know, and what it cannot under such conditions be taught. We shall have an inefficient nation—or, in the vernacular, "we shall spoil the ship for a ha'porth o' tar."

### THE VETERAN ARMY.

I am quite sure that Lord Knutsford can put his finger on scores, and probably hundreds, of nurses who are willing to work their fingers to the bone. If they are weary, they pretend that they are fresh. If the hospital is poor, they will willingly share its poverty. The same is true of domestic servants. Even in this revolutionary age there are armies of domestics who cling to the traditions of the past as to a religion. They regard themselves as a part of the family, and they rejoice and weep with the family in prosperity and in sorrow. It is admirable; it is magnificent. But, alas! it is not economics. The stern fact remains that such servants cannot be reproduced. Recruits are unobtainable. Your modern domestic wants references as to the character of her mistress, and in a very short time she will demand an architectural plan of the house where she is to live and work. We stand not where we used to stand. This is a part of the new heaven and the new earth.

### THE COLLEGE AND THE NURSE.

I suggest to the College of Nursing that a complete statement of fact should be obtained from hospital matrons throughout the United Kingdom as to the quality of applicants for recruiting the nursing profession. I am willing to believe that the big Metropolitan hospitals do not notice any decline. They always have the pick of the basket. But what of the others? There is no use in my hazarding an opinion. It would necessarily be circumscribed and narrow. The facts can be obtained only by means of a thorough inquiry. I have seen matrons grind their teeth and complain that nowadays the stuff is poor. "They are not ladies," is one way of putting it. "They are uneducated and careless," is another. I think that the College ought to find this out and make it plain whether or not the modern recruit to the nursing profession is up to the standard of twenty or even ten years ago. Within those years the world has progressed, especially in education. Your chimney-sweep of to-day is an intelligent reader of the daily newspaper. A generation ago he was illiterate and his daughter was content with being a charwoman. To-day she plays the piano. My observation, which may be narrow, leads me to the conclusion that the candidate probationer of 1920 is inferior, speaking generally, to her predecessors, and that the tendency is not forward. The heads of the profession seem to think, like the Minister of Education, that nursing cannot compete with other professions. Like the writer in the *Times*, I say that "it can and it must compete with other branches of the public service," and I hope that every nurse in the kingdom will endorse this.

The solution of the difficulty, which is very real and very substantial, lies in the field of economis. As the *Nursing Mirror* observed last week, "the tradition that the nurse is a creature partaking of the natures of an angel and a steam-crane dies hard." The tradition, indeed, appears to me to be embalmed. Even when it actually dies it keeps up the appearance of being alive. It is imperative that the truth should be ascertained. Is the potential angel-steam-crane nurse continuing to offer her services as probationer? The College of Nursing ought to be able to find out.

IERNE.

